# The Use of Neural Networks in the Analysis of Dual Adhesive Single Lap Joints Subjected to Uniaxial Tensile Test

**DOI:** 10.3390/ma14020419

**Published:** 2021-01-15

**Authors:** Jakub Gajewski, Przemysław Golewski, Tomasz Sadowski

**Affiliations:** 1 Department of Machine Design and Mechatronics, Faculty of Mechanical Engineering, Lublin University of Technology, Nadbystrzycka 36, 20-618 Lublin, Poland; 2 Department of Solid Mechanics, Faculty of Civil Engineering and Architecture, Lublin University of Technology, Nadbystrzycka 38, 20-618 Lublin, Poland; p.golewski@pollub.pl (P.G.); t.sadowski@pollub.pl (T.S.)

**Keywords:** dual adhesive, single lap joints, numerical modeling, artificial neural networks

## Abstract

Adhesive bonding are becoming increasingly important in civil and mechanical engineering, in the field of mobile applications such as aircraft or automotive. Adhesive joints offer many advantages such as low weight, uniform stress distribution, vibration damping properties or the possibility of joining different materials. The paper presents the results of numerical modeling and the use of neural networks in the analysis of dual adhesive single-lap joints subjected to a uniaxial tensile test. The dual adhesive joint was created through the use of adhesives with various parameters in terms of stiffness and strength. In the axis of the overlap, there was a point bonded joint characterized by greater stiffness and strength, and on the outside, there was a bonded joint limited by the edges of the overlap and characterized by lower stiffness and strength. It is an innovative solution for joining technology and the influence of such parameters as the thickness of one of the adherends, the radius of the point bonded joint and the material parameters of both adhesive layers were analyzed. The joint is characterized by a two-stage degradation process, i.e., after the damage of the rigid adhesive, the flexible adhesive ensures the integrity of the entire joint. For numerical modeling, the Finite Element Method (FEM) and cohesive elements was used, which served as input data to an Artificial Neural Network (ANN). The applied approach allowed the impact of individual parameters on the maximum force, initiation energy, and fracture energy to be studied.

## 1. Introduction

Adhesive joints are used in assembly technology in almost every field of engineering; it is increasingly used as an alternative method to welding, riveting and other conventional fasteners. In order to increase their strength and thus reduce the joining surface and/or the amount of adhesive, many techniques are used, such as: appropriate preparation of the substrate surface [1,2], chamfering the edges [3], selecting the shape of the overlap [4] or the use of hybrid joints [5,6].

One of the methods is also the use of connections of the “dual adhesive” or “mixed adhesive” type [7,8]. This method involves the use of two layers of adhesive with different properties in one joint. Layers of less stiff adhesive are placed at the ends of the overlap, and the stiff adhesive is used inside the overlap. The use of a flexible layer at the ends of the overlap allows for a significant reduction of stresses, thus increasing the strength of the entire joint. Intensive research into this type of connection is currently underway.

In [9], optimization of single lap joints of steel sheets was achieved through the selection of an appropriate set of adhesives. Four adhesives of different strengths and breaking strains were used. The best results were achieved by combining a very stiff with a very flexible and tough adhesive. An analytical solution was proposed for calculating the allowable breaking force of an adhesive connection.

A significant problem in dual adhesive joints is the phenomenon of mixing of both adhesives and the way they separate. Therefore, the authors in [10] carried out numerical simulations; two independent methodologies were proposed for selecting the intermediate material between the adhesive bands in mixed adhesive joints, attending to the singularity impact. Another approach is to allow the phenomenon of mixing. In the work [11], a Computational Fluid Dynamics (CFD) simulation concerning the flow of adhesive before curing and structural calculations after curing was performed. It has also been proposed to use a special nozzle for the simultaneous application of both adhesive layers.

In order to further optimize a mixed-adhesive connection and the separation of both adhesive layers, notches in the joined sheets can be used, an approach that was presented in [12]. Five types of connections were analyzed: a control without notches and test pieces with two, three, four and five notches. The aim was to find the optimal solution. The tangential stresses were found to be reduced by 34.5% and normal stresses by 26.4%. The best results were obtained for the model with five notches and a layer of epoxy and polyurethane adhesive. However, the disadvantage of this solution is the additional CNC machining operations required.

Depending on the choice of materials and adhesives, the advantages of dual adhesive joints may only become apparent when they are tested in a wider temperature range. The work [13] presents the results for single adhesive and dual adhesive joints using AV 138 (Huntsman Advanced Materials (Switzerland) GmbH Klybeckstrasse 200 CH - 4057 Basel, Switzerland) and SikaFast 5211 NT (Sika Deutschland GmbH Stuttgarter Str. 139, 72574 Bad Urach, Germany). The tests were carried out in the temperature range from −30 °C to 80 °C for both quasi-static and dynamic loads. In the temperature range from approx. −8 °C to 58 °C, higher breaking force was achieved for the dual adhesive joints; however, the absorbed energy was lower than for the single adhesive joints with the SikaFast 5211 NT flexible layer. Varying temperatures can occur in aviation and space applications. The authors in [14] glued ceramics to metal, which can be used in the installation of thermal barriers. The benefits of using a double adhesive layer were minimal. However, if we consider a temperature range from −65 °C to 100 °C, this type of layer allows the joint to work at the level of 50–60% strength in relation to the joint operating at an ambient temperature.

Due to the fact that in dual adhesive connections, both adhesives are mixed at contact, voids or weak bonds may occur [15]. Therefore, it is also important to conduct tests with the use of X-rays to analyze the internal structure [16].

Current research has been limited to analyzing a small number of commercial adhesives. However, in order to fully analyze the influence of material properties and geometric features, it is necessary to use neural networks [17,18,19]. Neural networks are a component machine learning, which can boost the efficacy of monitoring tools. Multi-layered neural network can be easily understood by an designers and engineers. These machine learning models can be directly deployed due to their increased universality and transparency compared to other methods used in exploratory data analysis and for making predictive models. Artificial neural networks are efficient computing and approximation models. The advantage of using neural networks models is also the ability to work with incomplete data. An analysis of the literature indicates that the research problem is topical. The use of artificial intelligence (AI) methods to analyze the strength of joints, including adhesive ones, is the subject of much research [20,21,22].

Numerical simulations were carried out in the Abaqus program; the results were used as input data to the neural network. The research was carried out on the novel concepts of joints with a rigid point adhesive joint surrounded by an elastic joint. The research results allowed the optimal range of parameters to be determined.

## 2. Dual Adhesive Model Description

**Figure 1:**
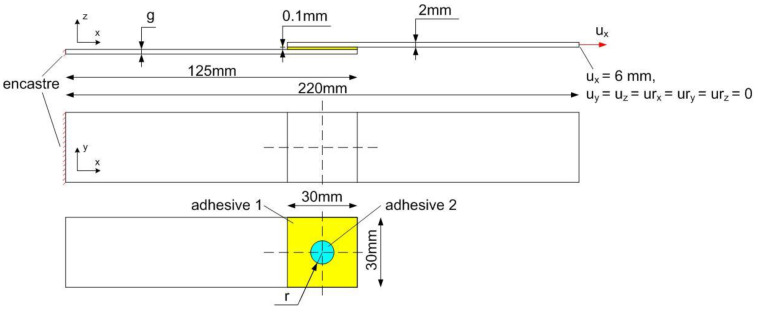
Single lap joint geometry.

Two types of adhesives are used in the single lap model: 1—an adhesive with lower stiffness and lower strength, and 2—an adhesive with higher stiffness and greater strength. This type of connection, called “dual adhesive” or “mixed adhesive,” is characterized by a two-step operation, which is described later in this article. In order to be able to properly select the strength of the joint and to influence its behavior after exceeding the load capacity, the proportions of the share of the adhesive surface 1 and 2 in the overlap should be selected appropriately. In the models considered, this was done by changing the radius “r” of the point adhesive joint. The second variable parameter was the thickness “g” of one of the adherends. The other dimensions were constant and are shown in Figure 1.

**Figure 2:**
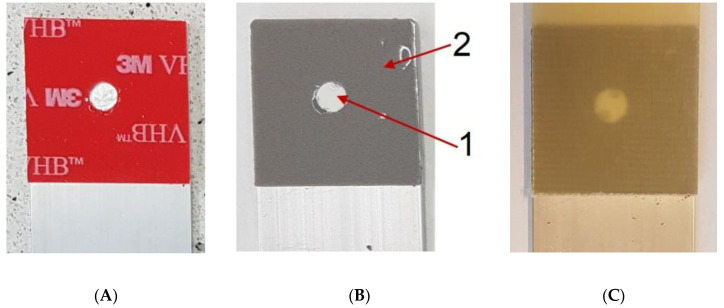
Example of dual adhesive sample preparation. (**A**) double side adhesive tape and liquid epoxy application; (**B**)removing of protective film and (**C**) adding a second adherend.

In addition to changes in the geometry, modifications were also made to the material properties of both adhesive joints. Data from [23] for such adhesives as Araldite 2015 and Araldite AV138 were used as input data for variable parameters of adhesive joints. The use of two liquid layers of adhesive is a technologically difficult issue, and currently the authors use only rectangular-shaped joints. To prevent the adhesives from mixing, different methods are used—e.g., by using silicone gaskets [24] or by adding fibers [25]. The technology of making this type of connection is not yet fully resolved. Therefore, the current work focuses only on the numerical model. However, the authors are working on the use of double-sided adhesive tape and liquid epoxy as a point joint. Figure 2 shows a practical embodiment of this type of connection. Initially, double-sided tape is applied to one of the joined parts and a hole is made with a die (Figure 2A). In the next step, the liquid epoxy is applied until the hole is filled and the protective film is removed (Figure 2B). Finally, the connection is made by adding a second adherend (Figure 2C). The use of double-sided tape as a layer of lower rigidity has the advantage that the joint formed by the epoxy point joint has the same geometry in each case, since no mixing occurs.

## 3. Numerical Modeling

Performing static, dynamic, or cyclic testing is always time-consuming considering samples and laboratory test stand preparation as well as equipment maintenance during the test. Therefore, with a large number of samples, it is necessary to use numerical methods [26].

In this work, 100 numerical simulations were carried out in the Abaqus 6.16 program (Dassault Systemes SIMULIA), which then served as input data to the neural network. Numerical modeling was performed in the Abaqus Explicit program. The joint geometry is shown in Figure 1. In the analyzed model, the following geometric variables were assumed:

- the thickness of one of the adherends “g” (2; 4; 6; 8; 10; 12; 14; 16; 18; 20) (mm),
- the radius of the point adhesive joint “r” (1; 2.25; 3.5; 4.75; 6; 7.25; 8.5; 9.75; 11; 12.25; 13.5; 14.75) (mm).

**Figure 3:**
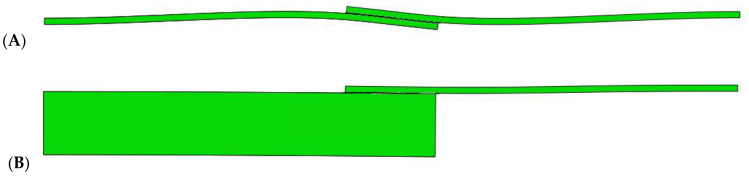
Deformation of single lap joints: (**A**) model 6 and (**B**) model 57.

The analysis of the influence of thickness is due to the fact that in a lap joint there is an eccentricity (distance between the lines of action of the load), which causes the joint to bend and additionally loads the adhesive joint. When the thickness of one or both of the adherends increases, the bending effect is reduced as shown in the Figure 3 for model 6 and model 57 for a similar load level. To better show the bending effect, the deformations of both models were scaled five times.

In addition to geometric changes, the properties of both adhesive joints were modified for Young’s modulus E, Kirchoff G modulus, shear strength and tensile strength. The scope of these changes is presented in Table 1. Parameter changes for 100 models are included in Appendix A.

The quadratic nominal stress criterion (QUADS) was used to describe the initiation of damage of the adhesive joint material. This criterion considers concurring quadratic ratios between nominal stress and allowable stress acting in different directions:

(1) $${\left(\frac{\sigma_{n}}{\sigma_{n}^{\max}}\right)}^{2}+{\left(\frac{\sigma_{t}}{\sigma_{t}^{\max}}\right)}^{2}+{\left(\frac{\sigma_{s}}{\sigma_{s}^{\max}}\right)}^{2}=1$$

where:

- *σ_n_* is the normal stress applied to the surface of the adhesive layer;
- *σ_t_* and *σ_s_* are the shear stress components along the adhesive layer;
- *σ*_nmax_, *σ*_tmax_ and *σ*_smax_ are the critical values of the normal and shear stress components corresponding to appropriate damage mode initiation.

Damage is assumed to initiate when the maximum nominal stress ratio reaches a value of one.

The elastic/plastic properties for the adherend material were adopted as for aluminum 2017 from TABAL LTD, Poland: Young’s modulus E = 72.5 GPa, Poisson ratio ν = 0.33, yield stress *σ*_y_ = 250 MPa, peak stress *σ*_u_ = 400 MPa and deformation at break A = 10%.

Before making the FEM mesh, a literature analysis was performed. J.J.M. Machado et al. [25] investigated the mesh refinement in the mixed adhesive single lap joints. The calculations were made for 4 mesh densities from 0.2 to 4 mm and from 1 to 10 elements along the thickness. The result clear show reduced mesh dependency of the results with mesh configuration, even with very coarse meshes. This demonstrates that cohesive element modelling is suitable for modelling of large and complex structures, as the need for refinement is minimal.

**Figure 4:**
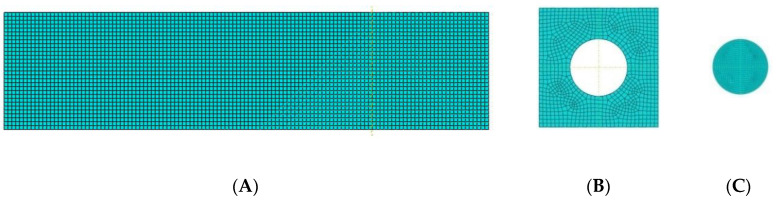
(**A**–**C**) Mesh of finite elements.

In the current model, the adherends was built on the basis of C3D8R (eight-node brick element with reduced integration) elements, the global size of the element was 1 mm and 4 elements along the thickness of the sheets. COH3D8 elements (eight-node three-dimensional cohesive element) were used for both adhesive layers, the global element size for the outer layer was 1 mm, and for the point joint 0.5 mm (Figure 4).

For all parts of the assembly, “tie” constraints were used, thereby removing all degrees of freedom between the surfaces in contact.

**Figure 5:**
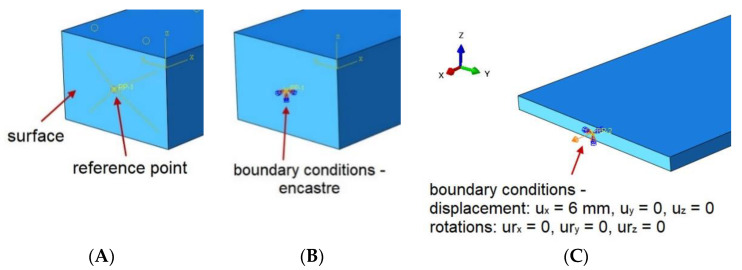
Boundary conditions. (**A**) Reference point connected to the surface; (**B**) Encastre boundary conditions and (**C**) Displacement boundary conditions.

In order to be able to read the reaction values and assign boundary conditions, the RP-1 and RP-2 reference points were created on the front surfaces of both laps (Figure 5).

**Figure 6:**
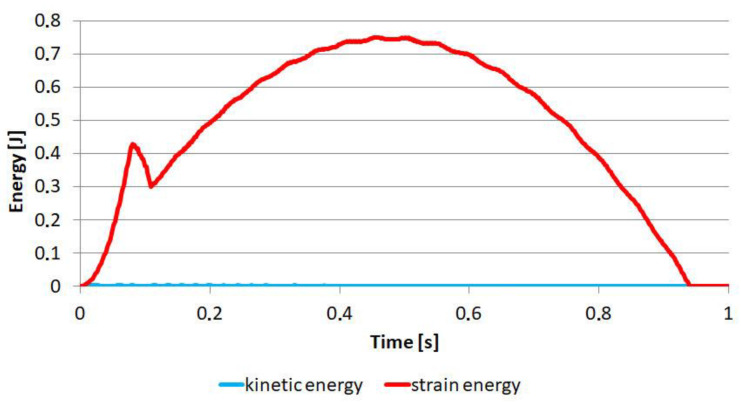
The change of energy over time.

The reference points were connected to the appropriate surfaces with a “coupling” type constraint, which allowed all the degrees of freedom to be transmitted (Figure 5A). One end of the sample was encastre (Figure 5B), while the other end could only move along the “x” axis up to 6 mm (Figure 5C). The simulations were carried out in Abaqus Explicit for a time of 1 s; therefore, it was also necessary to use mass scaling to shorten the calculation time. The target time increment was set to 2 × 10^−6^. After the simulation, the values of elastic energy and kinetic energy were controlled (Figure 6).

**Figure 7:**
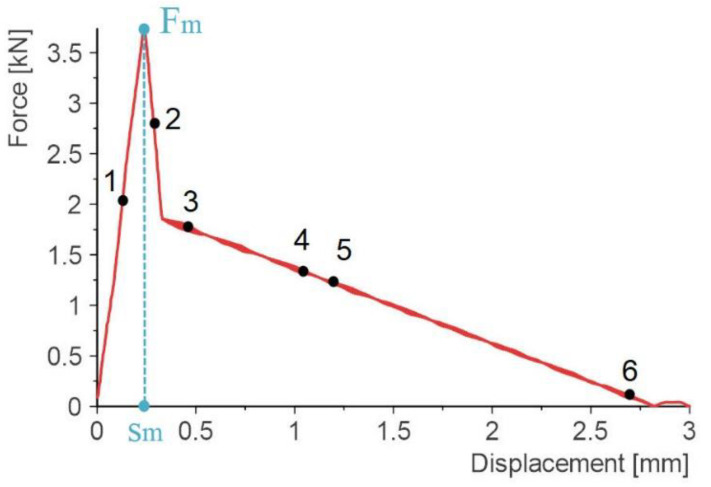
Force displacement graph for model 6.

An example force-displacement diagram is shown in Figure 7. For each model, the total energy required to fracture the sample and the damage initiation energy were calculated. The work done or the energy consumed for a given displacement is computed as:

(2) $$W_{\left(s\right)}=\int_{0}^{s}F_{\left(s\right)}ds$$

**Figure 8:**
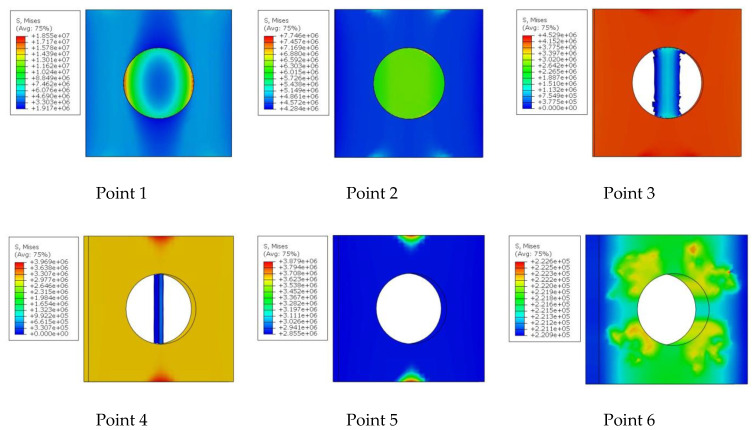
Degradation of adhesive layers for model 6 in [Pa] (1e6 = 1 × 10^6^ Pa).

The maximum force value (*F*_m_) and corresponding displacement (*s*_m_) were assumed as the limit point for calculating the initiation energy. Figure 7 additionally shows points from 1 to 6, which correspond to the stress fields in the adhesive layer in Figure 8.

In point 1, the load is transferred mainly through the point adhesive, which is responsible for the stiffness of the entire joint. A rigid and brittle connection fails with a slight displacement—in this case the maximum value was reached for a value of 0.25 mm. After reaching the maximum force, the stiffness of the point adhesive material gradually degrades. From point 3 onwards, the load is mainly carried by the outer joint. A small fragment in the axis of the overlap is still visible in point 4. In point 6, significant deformation of the outer joint is visible, which is damage for a displacement of 2.8 mm.

Until the external joint is damaged, the entire joint is an integral whole. As soon as the connection is relieved with a significant displacement of, e.g., 2 mm, both parts of the plate will still be connected. This type of connection can be used wherever it is required not only for a large force to be transmitted by the connection but also for a large displacement to be damaged, and thus a large fracture energy. Therefore, further work is planned for dynamic loads.

**Figure 9:**
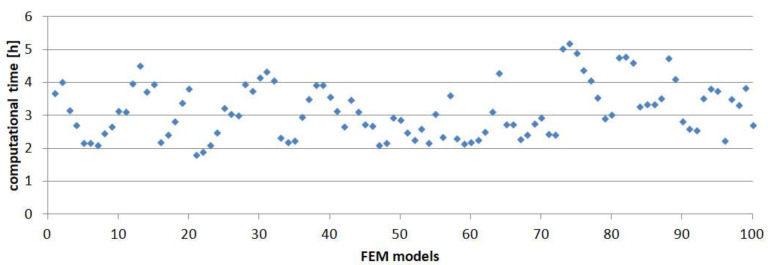
Computational time of FEM models.

The computational time for all models was presented in Figure 9.

Maximum and minimum values were, respectively, 5 h 11 min and 1 h 48 min; the average value for all models was 3 h 25 min. The differences in the computational time resulted mainly from the number of finite elements in the models due to the variable thickness of one of the adherends. The calculations were performed on a workstation equipped with two Intel(R) Xeon(R) 2.3 GHz processors (64 logical cores) and 256 GB of RAM.

## 4. Application of Neural Networks

The artificial neural network model was created based on analogies to biological counterparts. Neural networks are currently widely used in technical issues, among others [27,28,29]. They are a good solution for forecasting and regression problems. Neural networks are signal processing mathematical models. The main advantages of the neural network are that it works in conditions of incomplete information, it works automatically (does not require knowledge of the algorithm to solve the task), it can generalize (works in areas outside the input information).

Since each FEM simulation of the model take a long computational time, particularly, considering the computational costs [30], in order to be able to quickly simulate the adhesive joint parameters, the ANN modelling was used. In this research, the neural network is a model for predicting maximum connection strength, initiation energy and fracture energy. In numerical research a network with 10 input variables was applied. These are the parameters of adhesives and geometric dimensions of the connection (Table 2).

In the hidden network layer, 12 neurons were modelled. Output parameters are: (i) maximum force, (ii) initiation energy and (iii) fracture energy.

**Figure 10:**
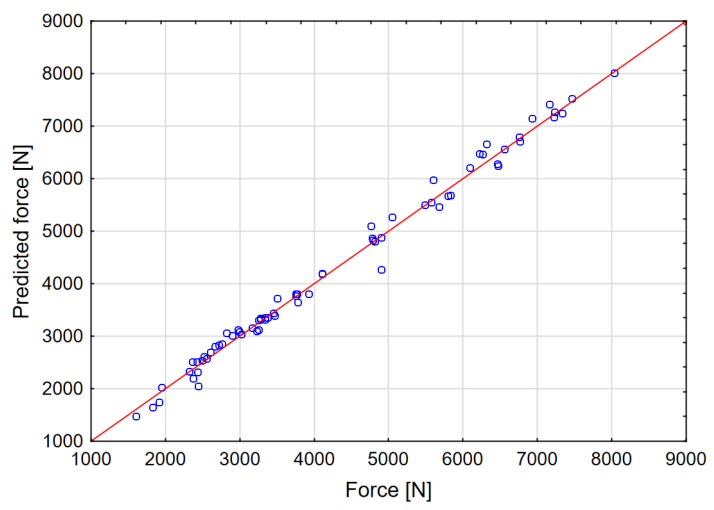
Actual versus predicted values: maximum force.

**Figure 11:**
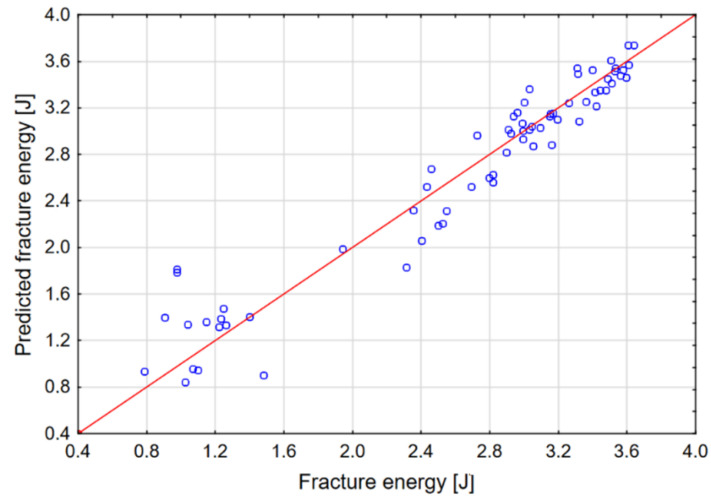
Actual versus predicted values: fracture energy.

**Figure 12:**
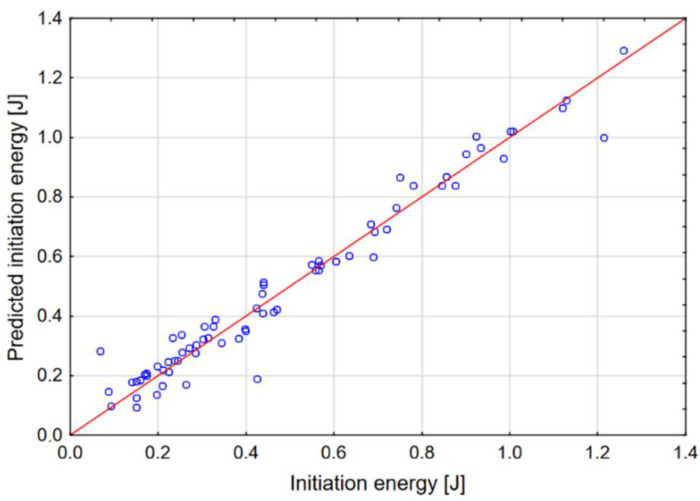
Actual versus predicted values: initiation energy.

The graphs in Figure 10, Figure 11 and Figure 12 show the dependence of the network output parameters on data from the numerical experiment.

The neural network is trained in such a way that its parameters are changed using the selected learning algorithm. The best known example of such an algorithm is the backpropagation algorithm. This algorithm, based on the collected data, modifies the weights and thresholds of the network in such a way as to ensure that the error made by the network (in this case, the prediction error) for all the data included in the training set is minimal. The data set included 100 cases from the numerical experiment. The data were classified as follows: 80% training, 10% test, 10% validation. The parameters and outputs of the network are presented in Table 3. It was found that a multilayer perceptron (MLP) provides the best quality, which is why this network model was qualified for calculation. The sigmoid network is the most frequently used artificial neural network. This network model has a multilayered placement of neurons; neurons calculate the sum of the inputs. These values are arguments of the function that calculates the output of the neurons. The predictive effectiveness of the neural network is based on data from the FEM experiment. It is calculated using the sum of squares error function. The predictive effectiveness of the MLP network for all output parameters was approx. 98%.

## 5. Discussion of the Results

The use of neural networks enables effective forecasting of force, initiation and fracture energy values. Determining the key parameters of the connection will allow its parameters to be optimized. Research has shown that the most important parameter for the strength of the joint is the size of the hole in which the more rigid adhesive is used. It has been shown that in the tested joint, in order to increase the maximum joint strength, it is advisable to use an internal adhesive with higher Young’s modulus values. Kirchoff G modulus values are important in relation to fracture energy.

Several conclusions emerge from the artificial neural network (ANN) sensitivity analysis. A sensitivity analysis makes it possible to distinguish important variables from those that are not relevant. The input variables in the analyzed case are not completely independent. The sensitivity analysis shows the loss we incur when we reject a particular variable. To carry out the analysis, the data is presented to the network repeatedly. In each test, all values of one variable are converted into missing data and the total error is calculated, similarly to the standard learning of a network. The key factor with respect to maximum force, fracture energy and initiation energy is the joint radius r. The value of radius r affects the results of numerical analyses in about 70%.

**Figure 13:**
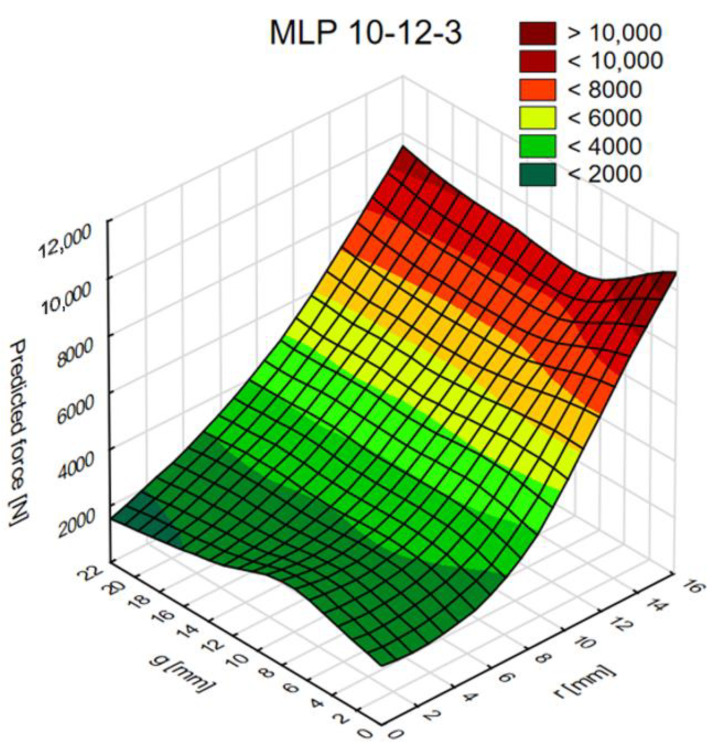
The graph of force values as a function of radius (r) and thickness (g).

**Figure 14:**
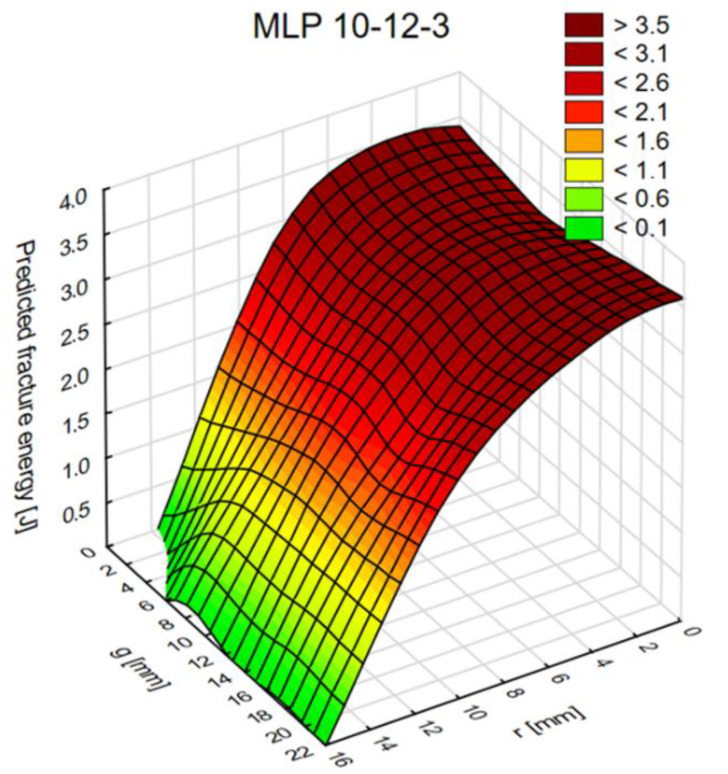
The graph of fracture energy values as a function of radius (r) and thickness (g).

**Figure 15:**
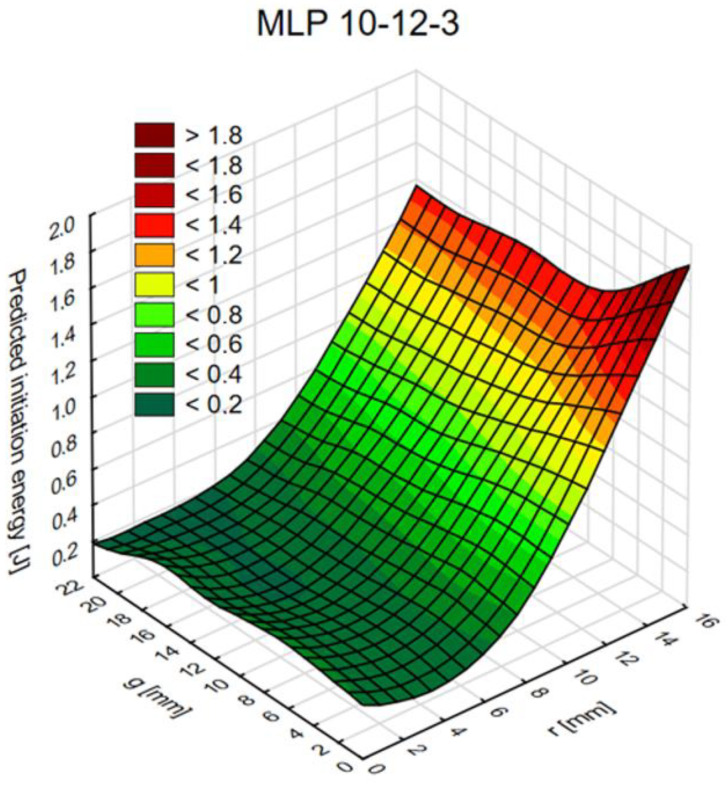
The graph of initiation energy values as a function of radius (r) and thickness (g).

Figure 13, Figure 14 and Figure 15 show diagrams of the dependence of ANN output parameters as a function of the diameter (radius r) and the thickness g of the connection.

**Figure 16:**
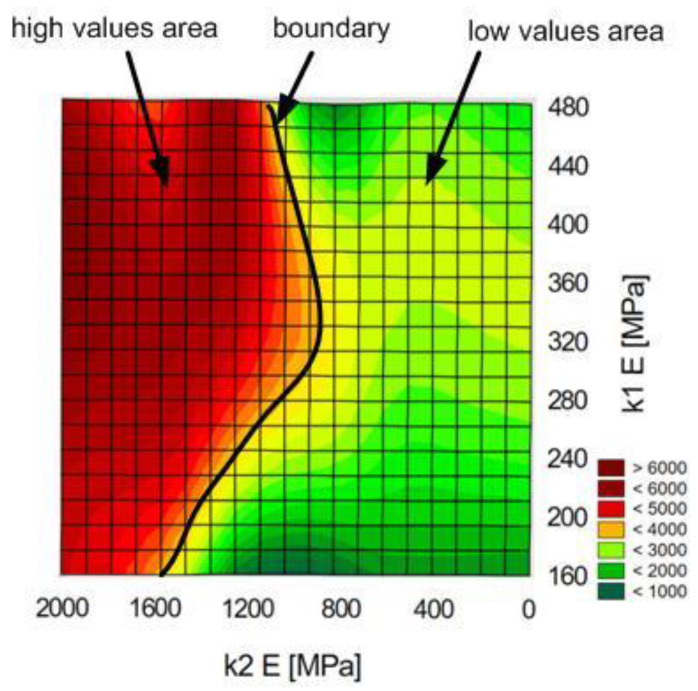
Diagram of force values [N] in function of Young’s moduli of both adhesives.

Separate tests were conducted to determine the most important input parameters of the network, in relation to individual output variables. The research assumed an average connection diameter value. The Young’s modulus (Figure 16) and the stress values of the adhesives used are the most important for the maximum joint force.

Figure 16 shows that the maximum force increases significantly for adhesive 2 Young’s modulus values above 1200 MPa.

**Figure 17:**
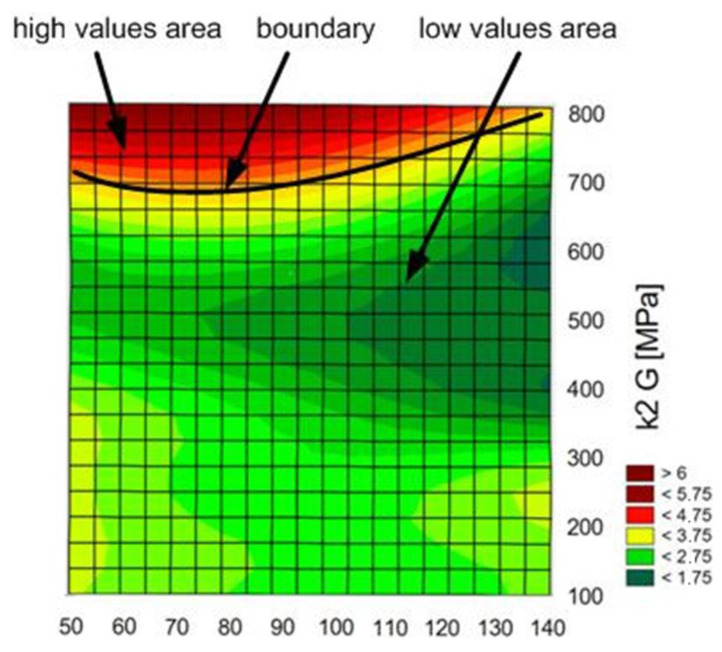
Diagram of fracture energy [J] in function of rigidity modulus of both adhesives.

For the forecasted energy values, the most important input variables are both adhesives’ rigidity moduli (Figure 17).

## 6. Conclusions

The strength of an adhesive joint is crucial for design and optimization of its mechanical response. Optimal selection of geometric parameters of the joint and properties of the adhesives improves safety and reduces costs. The application of ANN allows the assessment of the significance of individual connection parameters and their modification; the use of neural networks in joint design allows the correctness of numerical calculations to be verified. The Finite Element Method calculation time for different connection parameters is significantly reduced.

The tests conducted provide valuable information for future numerical and laboratory tests. An important conclusion concerns the thickness “g” of one of the adherends, the increase in which has little effect on changes in both the maximum force and the fracture energy. Moreover, testing the joints in the range of the value of the radius “r” from 2 to 6 mm is not advisable as the changes in the value of the maximum force and fracture energy are insignificant. Only in the “r” range from 8 to 16 mm does the sample response become more sensitive. The research also proves that dual adhesive joints are sensitive to the appropriate selection of the material parameters of both adhesives. In the work, the graphs define the limits for both the E and G module; when exceeded, the maximum force and fracture energy suddenly increase.

The presented work does not exhaust all the possibilities of using FEM and neural networks in relation to adhesive joints. Important parameters are also defects, excess adhesive or variable thickness of the adhesive. These types of parameters have a practical relationship with the strength of real structures subjected to the bonding technique. Knowledge about the size of the defect obtained, for example, from a tomograph, in conjunction with the knowledge resulting from the use of FEM and neural networks, can be used to assess the condition of the structure and prevent failures.

## Figures and Tables

**Table 1:** Ranges of changes in the parameters of dual adhesive joints.

	Modulus E (MPa)	Modulus G (MPa)	Shear Strength (MPa)	Tensile Strength (MPa)
Adhesive 1	185–436.22	56–132.05	1.7–4.01	2.2–5.19
Adhesive 2	716.73–1850	216.96–700	6.97–18	8.52–22

**Table 2:** Neural network input parameters.

Adhesive 1	Modulus E_1_ (MPa)	Modulus G_1_ (MPa)	Shear strength (k1) (MPa)	Tensile strength (k1) (MPa)
Adhesive 2	Modulus E_2_ (MPa)	Modulus G_2_ (MPa)	Shear strength (k2) (MPa)	Tensile strength (k2) (MPa)
Geometrical parameters	Radius r (mm)	Thickness g (mm)		

**Table 3:** Artificial neural networks operation parameters.

Network	Quality (Training)	Quality (Testing)	Quality (Validation)	Training Algorithm	Error Function	Activation (Hidden)	Activation (Output)
MLP 10–12–3	0980	0971	0981	Broyden-Fletcher–Goldfarb-Shanno	Sum ofsquares	Tanh	Linear

## Data Availability

The data presented in this study are available on request from the corresponding author.

## Appendix A

**Table A1:** Parameter values for each model.

			Adhesive 1				Adhesive 2			
No.	“r” (mm)	“g” (mm)	E (MPa)	G (MPa)	*σ* (MPa)	τ (MPa)	E (MPa)	G (MPa)	*σ* (MPa)	τ (MPa)
1	1	20	185	56	2.2	1.7	1213.79	367.42	14.43	11.81
2	2.25	16	203.50	61.60	2.42	1.87	983.17	297.61	11.69	9.57
3	3.5	12	223.85	67.76	2.66	2.06	1850.00	560.00	22.00	18.00
4	4.75	8	246.24	74.54	2.93	2.26	1092.41	330.67	12.99	10.63
5	6	4	270.86	81.99	3.22	2.49	884.85	267.85	10.52	8.61
6	7.25	2	297.94	90.19	3.54	2.74	1213.79	367.42	14.43	11.81
7	8.5	4	327.74	99.21	3.90	3.01	1850.00	560.00	22.00	18.00
8	9.75	6	360.51	109.13	4.29	3.31	1665.00	504.00	19.80	16.20
9	11	8	396.56	120.04	4.72	3.64	1498.50	453.60	17.82	14.58
10	12.25	10	436.22	132.05	5.19	4.01	1348.65	408.24	16.04	13.12
11	1	12	223.85	67.76	2.66	2.06	1213.79	367.42	14.43	11.81
12	2.25	14	297.94	90.19	3.54	2.74	1092.41	330.67	12.99	10.63
13	3.5	16	396.56	120.04	4.72	3.64	983.17	297.61	11.69	9.57
14	4.75	18	360.51	109.13	4.29	3.31	884.85	267.85	10.52	8.61
15	6	20	327.74	99.21	3.90	3.01	796.36	241.06	9.47	7.75
16	7.25	2	185.00	56.00	2.20	1.70	716.73	216.96	8.52	6.97
17	8.5	6	203.50	61.60	2.42	1.87	1092.41	330.67	12.99	10.63
18	9.75	10	223.85	67.76	2.66	2.06	884.85	267.85	10.52	8.61
19	11	14	246.24	74.54	2.93	2.26	1665.00	504.00	19.80	16.20
20	12.25	18	270.86	81.99	3.22	2.49	716.73	216.96	8.52	6.97
21	1	2	297.94	90.19	3.54	2.74	645.06	195.26	7.67	6.28
22	2.25	4	327.74	99.21	3.90	3.01	580.55	175.73	6.90	5.65
23	3.5	6	360.51	109.13	4.29	3.31	522.49	158.16	6.21	5.08
24	4.75	8	396.56	120.04	4.72	3.64	470.25	142.34	5.59	4.58
25	6	10	436.22	132.05	5.19	4.01	423.22	128.11	5.03	4.12
26	7.25	12	327.74	99.21	3.90	3.01	380.90	115.30	4.53	3.71
27	8.5	14	360.51	109.13	4.29	3.31	342.81	103.77	4.08	3.34
28	9.75	16	185.00	56.00	2.20	1.70	1850.00	560.00	22.00	18.00
29	11	18	203.50	61.60	2.42	1.87	1665.00	504.00	19.80	16.20
30	12.25	20	223.85	67.76	2.66	2.06	1498.50	453.60	17.82	14.58
31	1	20	246.24	74.54	2.93	2.26	1092.41	330.67	12.99	10.63
32	2.25	12	270.86	81.99	3.22	2.49	1092.41	330.67	12.99	10.63
33	3.5	6	297.94	90.19	3.54	2.74	1850.00	560.00	22.00	18.00
34	4.75	2	327.74	99.21	3.90	3.01	716.73	216.96	8.52	6.97
35	6	4	360.51	109.13	4.29	3.31	983.17	297.61	11.69	9.57
36	7.25	8	396.56	120.04	4.72	3.64	796.36	241.06	9.47	7.75
37	8.5	12	436.22	132.05	5.19	4.01	1498.50	453.60	17.82	14.58
38	9.75	20	297.94	90.19	3.54	2.74	1348.65	408.24	16.04	13.12
39	11	18	327.74	99.21	3.90	3.01	884.85	267.85	10.52	8.61
40	12.25	16	360.51	109.13	4.29	3.31	1213.79	367.42	14.43	11.81
41	1	10	396.56	120.04	4.72	3.64	983.17	297.61	11.69	9.57
42	2.25	6	396.56	120.04	4.72	3.64	1498.50	453.60	17.82	14.58
43	3.5	18	185.00	56.00	2.20	1.70	1348.65	408.24	16.04	13.12
44	4.75	14	203.50	61.60	2.42	1.87	884.85	267.85	10.52	8.61
45	6	10	223.85	67.76	2.66	2.06	1665.00	504.00	19.80	16.20
46	7.25	2	246.24	74.54	2.93	2.26	716.73	216.96	8.52	6.97
47	8.5	4	270.86	81.99	3.22	2.49	1092.41	330.67	12.99	10.63
48	9.75	4	297.94	90.19	3.54	2.74	1213.79	367.42	14.43	11.81
49	11	12	327.74	99.21	3.90	3.01	983.17	297.61	11.69	9.57
50	12.25	10	360.51	109.13	4.29	3.31	1665.00	504.00	19.80	16.20
51	1	8	396.56	120.04	4.72	3.64	1498.50	453.60	17.82	14.58
52	2.25	2	396.56	120.04	4.72	3.64	1348.65	408.24	16.04	13.12
53	3.5	8	203.50	61.60	2.42	1.87	796.36	241.06	9.47	7.75
54	4.75	4	223.85	67.76	2.66	2.06	1850.00	560.00	22.00	18.00
55	6	12	246.24	74.54	2.93	2.26	1213.79	367.42	14.43	11.81
56	7.25	2	223.85	67.76	2.66	2.06	1665.00	504.00	19.80	16.20
57	8.5	20	246.24	74.54	2.93	2.26	983.17	297.61	11.69	9.57
58	9.75	6	270.86	81.99	3.22	2.49	1850.00	560.00	22.00	18.00
59	11	2	327.74	99.21	3.90	3.01	1498.50	453.60	17.82	14.58
60	12.25	4	360.51	109.13	4.29	3.31	1348.65	408.24	16.04	13.12
61	1	4	294	81.99	3.51	2.71	1204.00	367.42	14.33	11.72
62	2.25	8	294	90.19	3.51	2.71	1204.00	297.61	14.33	11.72
63	3.5	14	294	56	3.51	2.71	1204.00	560.00	14.33	11.72
64	4.75	20	294	61.60	3.51	2.71	1204.00	330.67	14.33	11.72
65	6	10	294	67.76	3.51	2.71	1204.00	267.85	14.33	11.72
66	7.25	2	294	74.54	3.51	2.71	1204.00	367.42	14.33	11.72
67	8.5	4	294	81.99	3.51	2.71	1204.00	560.00	14.33	11.72
68	9.75	6	294	90.19	3.51	2.71	1204.00	504.00	14.33	11.72
69	11	8	294	99.21	3.51	2.71	1204.00	453.60	14.33	11.72
70	12.25	10	294	109.13	3.51	2.71	1204.00	408.24	14.33	11.72
71	13.5	12	294	120.04	3.51	2.71	1204.00	580.00	14.33	11.72
72	14.75	14	294	132.05	3.51	2.71	1204.00	600.00	14.33	11.72
73	1	16	294	90.19	3.51	2.71	1204.00	620.00	14.33	11.72
74	2.25	18	294	81.99	3.51	2.71	1204.00	640.00	14.33	11.72
75	3.5	20	294	56	3.51	2.71	1204.00	660.00	14.33	11.72
76	4.75	16	294	120.04	3.51	2.71	1204.00	680.00	14.33	11.72
77	6	12	294	56	3.51	2.71	1204.00	367.42	14.33	11.72
78	7.25	8	294	61.60	3.51	2.71	1204.00	297.61	14.33	11.72
79	8.5	4	294	67.76	3.51	2.71	1204.00	560.00	14.33	11.72
80	9.75	2	294	74.54	3.51	2.71	1204.00	330.67	14.33	11.72
81	11	20	294	81.99	3.51	2.71	1204.00	267.85	14.33	11.72
82	12.25	16	294	90.19	3.51	2.71	1204.00	367.42	14.33	11.72
83	13.5	18	294	99.21	3.51	2.71	1204.00	560.00	14.33	11.72
84	14.75	4	294	109.13	3.51	2.71	1204.00	504.00	14.33	11.72
85	1	8	294	120.04	3.51	2.71	1204.00	453.60	14.33	11.72
86	2.25	12	294	132.05	3.51	2.71	1204.00	408.24	14.33	11.72
87	3.5	10	294	90.19	3.51	2.71	1204.00	580.00	14.33	11.72
88	4.75	20	294	81.99	3.51	2.71	1204.00	620.00	14.33	11.72
89	6	12	294	56	3.51	2.71	1204.00	660.00	14.33	11.72
90	7.25	2	294	120.04	3.51	2.71	1204.00	700.00	14.33	11.72
91	8.5	18	294	56	3.51	2.71	1204.00	367.42	14.33	11.72
92	9.75	6	294	61.60	3.51	2.71	1204.00	297.61	14.33	11.72
93	11	12	294	67.76	3.51	2.71	1204.00	560.00	14.33	11.72
94	12.25	20	294	74.54	3.51	2.71	1204.00	330.67	14.33	11.72
95	13.5	14	294	81.99	3.51	2.71	1204.00	267.85	14.33	11.72
96	14.75	4	294	90.19	3.51	2.71	1204.00	367.42	14.33	11.72
97	11	18	294	99.21	3.51	2.71	1204.00	560.00	14.33	11.72
98	12.25	10	294	109.13	3.51	2.71	1204.00	504.00	14.33	11.72
99	13.5	14	294	120.04	3.51	2.71	1204.00	453.60	14.33	11.72
100	14.75	8	294	132.05	3.51	2.71	1204.00	408.24	14.33	11.72
